# Gut Microbiota-Directed Interventions in Type 2 Diabetes: A Systematic Review of Clinical Outcomes and Complication Risk

**DOI:** 10.7759/cureus.95045

**Published:** 2025-10-21

**Authors:** Mustafeez Ur Rehman, Hadia Saeed, Osman Omer, Shahbaz Tashfeen

**Affiliations:** 1 Internal Medicine, Manchester University National Health Service (NHS) Foundation Trust, Manchester, GBR; 2 Emergency Department, King's College Hospital, London, GBR; 3 Integrative Medicine, Prince Mohammed Bin Abdulaziz Hospital, Madinah, SAU; 4 Internal Medicine, Nishtar Medical University, Multan, PAK

**Keywords:** cardiometabolic risk, complications, gut microbiota, insulin resistance, personalized nutrition, prebiotics, probiotics, type 2 diabetes

## Abstract

Type 2 diabetes mellitus is increasingly recognized as a disorder not only of glucose metabolism but also of gut microbial imbalance, with emerging evidence suggesting a bidirectional link between microbiome composition and metabolic dysfunction. Recent randomized controlled trials and dietary interventions highlight that specific microbial taxa, such as butyrate-producing bacteria and *Akkermansia muciniphila*, play crucial roles in regulating insulin sensitivity, lipid metabolism, and systemic inflammation. Modulation of the gut-metabolic axis through probiotics, prebiotics, dietary strategies, and pharmacological agents demonstrates promising effects on glycemic control, cardiometabolic risk reduction, and attenuation of diabetes-related complications, though results vary across populations and intervention types. The variability in outcomes underscores the importance of personalized approaches, where baseline microbiota signatures may dictate therapeutic response. Despite encouraging findings, many studies remain limited by short duration, small sample size, and heterogeneity in microbiome analysis methods. This review synthesizes current evidence, highlights mechanistic insights linking microbial shifts to metabolic benefits, and identifies gaps in the literature. By doing so, it emphasizes the potential of microbiome-directed therapies as adjunctive strategies in the prevention and management of type 2 diabetes and its complications.

## Introduction and background

Type 2 diabetes mellitus (T2DM) is one of the most prevalent metabolic disorders worldwide, accounting for more than 90% of diabetes cases. Its hallmark features include chronic hyperglycemia, insulin resistance, and progressive β-cell dysfunction, which together increase the risk of long-term complications such as cardiovascular disease, nephropathy, neuropathy, and retinopathy [1,2]. Despite advances in pharmacotherapy and lifestyle interventions, the global burden of T2DM continues to rise, underscoring the need to explore novel determinants of disease development and progression [3].

In recent years, the gut microbiome has emerged as a key player in metabolic health. The human intestinal microbiota functions as a metabolic “organ,” influencing host physiology through nutrient absorption, immune modulation, bile acid metabolism, and production of bioactive metabolites such as short-chain fatty acids (SCFAs), trimethylamine-N-oxide (TMAO), and succinate [4,5]. Alterations in microbiome composition and diversity, collectively termed dysbiosis, have been consistently associated with obesity, insulin resistance, and T2DM. Dysbiosis may exacerbate systemic inflammation, impair insulin signaling, and contribute to the development of both microvascular and macrovascular complications [6].

Multiple interventional studies have begun to evaluate whether targeted modulation of the gut microbiome, through dietary interventions, probiotics, prebiotics, synbiotics, fecal microbiota transplantation (FMT), and antidiabetic pharmacotherapies, can improve insulin sensitivity and reduce cardiometabolic risk [7]. For example, supplementation with Akkermansia muciniphila, use of specific probiotics, and diets enriched in fiber or omega-3 fatty acids have demonstrated beneficial effects on glucose metabolism and inflammatory pathways. Similarly, widely used antidiabetic agents such as metformin and SGLT2 inhibitors appear to exert part of their therapeutic effects through microbiome-mediated mechanisms.

The objective of this systematic review is to synthesize and critically evaluate clinical evidence on gut microbiota-directed interventions in adults with type 2 diabetes, with a dual focus on glycemic outcomes and diabetes-related complications. Unlike previous reviews that examined isolated probiotic or dietary effects, this study integrates findings across diverse interventional strategies, including dietary, pharmacological, and personalized nutrition approaches, to provide a comprehensive overview of how modulation of the gut-metabolic axis influences both metabolic control and complication risk. By highlighting mechanistic insights and identifying evidence gaps, this review aims to clarify the clinical potential and limitations of microbiome-targeted therapies in the context of precision diabetes management [8].

## Review

Materials and methods

Study Design and Protocol Registration

This study was conducted as a systematic review in accordance with the Preferred Reporting Items for Systematic Reviews and Meta-Analyses (PRISMA) 2020 guidelines [9]. The review protocol was designed a priori to ensure methodological transparency and consistency. The primary objective was to evaluate the role of gut microbiome composition in insulin resistance and the development of complications in type 2 diabetes mellitus (T2DM). The review incorporated randomized controlled trials (RCTs), pilot studies, and dietary intervention trials that directly assessed the impact of microbiota-targeted therapies, dietary modulation, or pharmacological interventions on metabolic outcomes.

Eligibility Criteria (PICO Framework)

The inclusion criteria were defined using the PICO framework [10]. The Population (P) included adult individuals with type 2 diabetes, obesity, metabolic syndrome, or insulin resistance. The Interventions (I) considered were microbiome-directed therapies, including probiotics, prebiotics, synbiotics, dietary modifications, microbiota-targeted personalized nutrition, or pharmacological interventions with a demonstrated effect on the gut microbiome. The Comparators (C) comprised placebo, standard care, conventional dietary regimens, or alternative interventions not targeting the gut microbiome. The Outcomes (O) of interest included glycemic parameters (glycated hemoglobin A1c (HbA1c), fasting glucose, postprandial glucose, insulin sensitivity indices), cardiometabolic markers (lipid profile, blood pressure, inflammatory markers), diabetes-related complications (neuropathy, vasculopathy), and gut microbiota composition or diversity. Only randomized controlled trials or controlled dietary interventions published in peer-reviewed journals were eligible. Studies involving pediatric populations, animal models, or those without primary outcomes related to both gut microbiota and metabolic health were excluded.

Information Sources and Search Strategy

A comprehensive literature search was conducted across PubMed, Embase, Web of Science, and Cochrane Central Register of Controlled Trials from inception until September 2024. Search terms were developed in consultation with controlled vocabulary (MeSH) and keywords, combining terms such as “type 2 diabetes,” “insulin resistance,” “gut microbiota,” “microbiome,” “complications,” “diet,” “probiotics,” “prebiotics,” and “cardiometabolic risk.” Boolean operators and truncation were applied to maximize retrieval. The reference lists of relevant reviews and included studies were also screened to capture additional eligible trials. Only studies published in English were considered.

Study Selection and Data Extraction

Titles and abstracts retrieved from the initial search were independently screened by two reviewers for eligibility. Full-text assessment was performed for all potentially relevant studies. Discrepancies were resolved through discussion or by a third reviewer when necessary. Data were systematically extracted into a predesigned form, capturing study characteristics (author, year, country, design, sample size), participant demographics, details of intervention and comparator arms, duration of follow-up, and primary and secondary outcomes. Key findings were summarized for narrative synthesis.

Risk of Bias Assessment

The quality and risk of bias of included RCTs were assessed using the Cochrane Risk of Bias 2.0 tool (RoB 2) [11]. The tool evaluates five domains: randomization process, deviations from intended interventions, missing outcome data, measurement of the outcome, and selection of the reported result. Each domain was judged as low risk, some concerns, or high risk, with an overall risk of bias rating for each study. This assessment was conducted independently by two reviewers, with disagreements resolved by consensus.

Data Synthesis and Analysis

Given the heterogeneity in interventions and outcomes across studies, a meta-analysis was not performed, and results were synthesized narratively. A structured summary of study designs, populations, interventions, comparators, outcomes, and key findings was compiled in tabular form. Particular emphasis was placed on linking microbiota composition and functional shifts with metabolic outcomes, including glycemic control, insulin resistance, lipid regulation, and diabetes-related complications. Where applicable, trends and mechanistic insights into microbiome-host interactions were highlighted to provide context for clinical implications and future research directions.

Results

Study Selection Process

**Figure 1:**
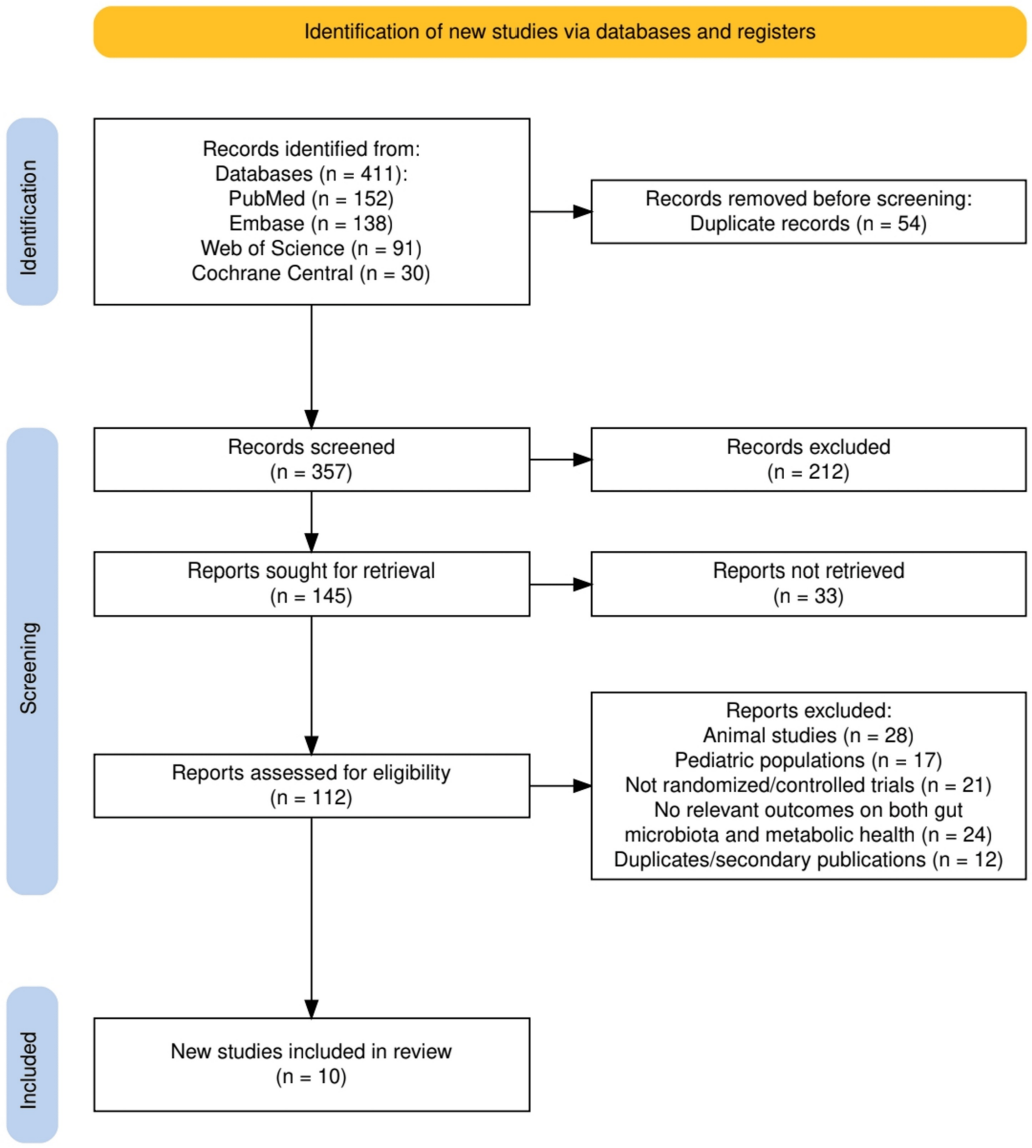
The PRISMA flowchart represents the study selection process PRISMA: Preferred Reporting Items for Systematic Reviews and Meta-Analyses.

A total of 411 records were identified through database searches, including PubMed (n = 152), Embase (n = 138), Web of Science (n = 91), and Cochrane Central (n = 30). After removal of 54 duplicates, 357 records were screened by title and abstract, of which 212 were excluded for irrelevance. Of the remaining 145 reports sought for retrieval, 33 could not be accessed, leaving 112 full-text articles assessed for eligibility. Following detailed evaluation, 28 animal studies, 17 involving pediatric populations, 21 without randomized or controlled trial design, 24 lacking outcomes related to both gut microbiota and metabolic health, and 12 duplicate or secondary publications were excluded. Ultimately, 10 studies met the inclusion criteria and were synthesized for this systematic review. The whole screening process is illustrated in Figure 1.

Characteristics of the Selected Studies

The 10 randomized controlled and controlled dietary intervention trials included in this review demonstrated a diverse range of microbiome-targeted approaches in type 2 diabetes and metabolic syndrome. As summarized in Table 1, interventions ranged from probiotic and synbiotic supplementation (e.g., *Akkermansia muciniphila*, *Bifidobacterium breve* with berberine) to pharmacological agents (empagliflozin), functional foods (white bean extract, enriched seafood sticks, whole-grain rye), and personalized nutrition guided by microbiome profiling. Across studies, improvements were consistently observed in glycemic parameters, insulin sensitivity, lipid metabolism, and cardiometabolic risk markers, with several trials also linking these outcomes to shifts in gut microbial diversity and composition. Collectively, these findings highlight both the therapeutic potential and heterogeneity of microbiome-based strategies in modulating insulin resistance and complication risk in type 2 diabetes.

**Table 1:** Summary of randomized controlled and dietary intervention trials This summary evaluates gut microbiota-directed therapies and their effects on metabolic and cardiometabolic outcomes in type 2 diabetes mellitus and related metabolic disorders. RCT: Randomized controlled trial, BMI: Body mass index, LDLc/LDL-C: Low-density lipoprotein cholesterol, BBR: Berberine, FFA: Free fatty acids, T2DM: Type 2 diabetes mellitus, PPT: Postprandial-targeting, MED/Med: Mediterranean diet, CGM: Continuous glucose monitoring, HbA1c: Glycated hemoglobin, 16S rRNA: 16S ribosomal ribonucleic acid, LC-MS: Liquid chromatography–mass spectrometry, HOMA-IR: Homeostatic model assessment for insulin resistance, PP: Pulse pressure, CVD: Cardiovascular disease, BP: Blood pressure, CRP: C-reactive protein, WCBE: White common bean extract, TCSS: Toronto Clinical Scoring System, SNCV: Sural nerve conduction velocity, SCFA: Short-chain fatty acid, MUFA: Monounsaturated fatty acid, LFHCC: Low-fat, high-complex carbohydrate, OGTT: Oral glucose tolerance test, WG: Whole grain, FC: Fold change, CFU: Colony-forming units, CG: Control group, ASV: Amplicon sequence variant, E. coli: Escherichia coli, A. muciniphila: Akkermansia muciniphila, B. animalis subsp. lactis: Bifidobacterium animalis subspecies lactis, B. breve: Bifidobacterium breve, B. angulatum: Bifidobacterium angulatum, L. brevis: Lactobacillus brevis, n: Sample size (number of participants), mmHg: Millimeters of mercury, mg/dL: Milligrams per deciliter, mmol/L: Millimoles per liter, g/day: Grams per day, mg/day: Milligrams per day, ↑: Increase, ↓: Decrease, PREMOTE: Trial acronym as reported by the authors (full expansion not provided in the table), CECT: Colección Española de Cultivos Tipo (Spanish Type Culture Collection), subsp.: Subspecies, vs.: Versus.

Authors (Year)	Study Design	Population (P)	Intervention (I)	Comparator (C)	Outcomes (O)	Key Findings
Depommier et al., 2019 [12]	RCT (double-blind, placebo-controlled, pilot)	40 overweight/obese insulin-resistant adults (32 completed)	Oral supplementation with live or pasteurized Akkermansia muciniphila for 3 months	Placebo	Primary: Safety, tolerability, metabolic parameters (insulin resistance, lipids, adiposity, BMI). Secondary: gut barrier function, gut microbiota composition	Pasteurized A. muciniphila improved insulin sensitivity (+28.6%), reduced insulinemia (-34%) and cholesterol (-8.7%); slight reduction in weight, fat mass, and hip circumference. Safe and well-tolerated.
Wang et al., 2022 [13]	Multicenter, double-blind, placebo-controlled RCT	365 patients with type 2 diabetes (PREMOTE study)	Combined berberine (BBR) + probiotics (Bifidobacterium breve) for 3 months	Placebo; BBR alone; Probiotic alone	Primary: Postprandial lipidemia (total cholesterol, LDLc). Secondary: gut microbiota composition, lipidomic metabolites, mechanistic gene expression	Combination (Prob+BBR) significantly reduced postprandial cholesterol and LDLc vs. control and single interventions; linked to increased B. breve activity and FFA metabolism; suggests synergistic hypolipidemic and microbiome-mediated effect.
Rein et al., 2022 [14]	Randomized crossover pilot trial (with six-month extension)	23 adults with newly diagnosed T2DM (mean age 53.5 ± 8.9 years, 48% male)	Personalized postprandial-targeting (PPT) diet using machine learning integrating microbiome & clinical features	Mediterranean-style diet (MED)	CGM-based glycemic measures, HbA1c, fasting glucose, triglycerides, gut microbiome shifts	PPT diet significantly lowered postprandial glucose response, mean glucose, and time >140 mg/dl vs. MED diet. Long-term PPT reduced HbA1c (−0.39%), fasting glucose (−16.4 mg/dl), triglycerides (−49 mg/dl), with 61% achieving diabetes remission. Clinical improvements linked to microbiome changes.
Deng et al., 2022 [15]	Randomized, open-label, two-arm clinical trial (three months)	76 treatment-naïve T2DM patients with cardiovascular risk factors (Empagliflozin n=40; Metformin n=36)	Empagliflozin 10 mg/day	Metformin 1700 mg/day	HbA1c, glucose metabolism, cardiovascular risk factors, gut microbiota (16S rRNA), plasma metabolites (LC-MS)	Both groups reduced HbA1c, but only empagliflozin improved cardiovascular risk factors. Empagliflozin reshaped gut microbiota (↑ Roseburia, Eubacterium, Faecalibacterium; ↓ Escherichia-Shigella, Bilophila, Hungatella) and altered plasma metabolites (↑ sphingomyelin; ↓ glycochenodeoxycholate, cis-aconitate, uric acid). Suggests cardiovascular benefit linked to microbiota/metabolite shifts.
Companys et al., 2022 [16]	Randomized, double-blind, placebo-controlled parallel trial (12 weeks + acute 4h test)	120 abdominally obese adults	50 g/day enriched seafood sticks containing heat-inactivated B. animalis subsp. lactis CECT8145 (10¹⁰ CFU), 370 mg/day omega-3, and 1.7 g/day inulin	Placebo seafood sticks (50 g/day)	Insulin, HOMA-IR, pulse pressure (PP), postprandial triglycerides, gut microbiota composition	Significant ↓ insulin (-5.25 mg/dL) and HOMA-IR (-1.33). In women, ↓ PP (-4.69 mmHg). Acute study: lower rise in postprandial triglycerides vs placebo (+23.9 vs +49.0 mg/dL). Gut microbiota: ↓ Alistipes finegoldii and Ruminococcaceae linked to glycemic improvement; ↓ Prevotella 9-ASV0283 and Christensenellaceae linked to PP reduction. Suggests protection against T2DM and CVD risk.
Kallapura et al., 2024 [17]	Prospective, open-label, randomized, controlled, proof-of-concept study (three months)	30 participants with T2DM and hyperlipidemia (Test arm n=15; Control arm n=15)	Microbiome-based personalized nutrition guided by BugSpeaks® microbiota profiling (whole genome shotgun sequencing → tailored diet)	Standard diabetic nutritional guidance	HbA1c, BP, CRP, gut microbiota diversity and composition	Test arm: HbA1c ↓ from 8.3 → 6.67 (p<0.001); systolic BP ↓ 5% (subgroup with >130 mmHg: ↓ 14%); CRP ↓ 19.5%. Gut microbiota showed ↑ beneficial species (Phascolarctobacterium succinatutens, B. angulatum, L. brevis) and ↓ potentially harmful species (A. finegoldii, S. faecalis). Significant ↑ Shannon diversity (p<0.05). Overall: improved glycemia, BP, inflammation linked to microbiota modulation.
Feng et al., 2022 [18]	Randomized, double-blinded, placebo-controlled clinical trial (four months)	90 T2DM patients (intensive phase) → 55 continued in maintenance phase	White Common Bean Extract (WCBE, 1.5 g before meals, α-amylase inhibitor) + normal carbohydrate intake	Placebo (1.5 g maltodextrin)	HbA1c, diabetic neuropathy (TCSS), sural sensory nerve conduction velocity, gut microbiota composition	WCBE group: HbA1c ↓ by 0.72% (p<0.05); fewer cases of diabetic peripheral neuropathy; sural SNCV improved (vs. decline in placebo). Gut microbiota: ↑ Bifidobacterium, Faecalibacterium, Anaerostipes; ↓ Weissella, Klebsiella, Cronobacter, Enterobacteriaceae. SCFA-producers enrichment linked to benefits. Overall: WCBE improved glycemic control and attenuated diabetic complications via microbiota modulation.
Haro et al., 2016 [19]	Randomized, open-label, controlled dietary intervention (one year)	20 obese men (from the CORDIOPREV study, with coronary heart disease)	Mediterranean diet (35% fat, 22% MUFA)	Low-fat, high-complex carbohydrate (LFHCC) diet (28% fat, 12% MUFA)	Gut microbiota composition, fecal and plasma metabolome, insulin sensitivity markers	LFHCC diet: ↑ Prevotella, ↓ Roseburia. Mediterranean diet: ↓ Prevotella, ↑ Roseburia & Oscillospira. Both diets: ↑ beneficial species (Parabacteroides distasonis with Med; Faecalibacterium prausnitzii with LFHCC). Microbiota shifts correlated with metabolomic changes (amino acids, peptides, sphingolipids). Overall, both diets improved gut microbial balance linked to improved insulin sensitivity and protection against T2DM development.
Balfegó et al., 2016 [20]	Pilot randomized controlled trial (six months)	35 drug-naïve patients with type 2 diabetes	Standard T2DM diet + 100 g sardines, 5 days/week	Standard T2DM diet (control)	Glycemic control (HbA1c, glucose, insulin, HOMA-IR), adiponectin, inflammatory markers, erythrocyte membrane fatty acid composition, gut microbiota composition	Both groups ↓ fasting insulin and HOMA-IR (improved insulin sensitivity). Only sardine group ↑ adiponectin (+40.7%, P = 0.04) and omega-3 index (+2.6% vs +0.6% in CG, P = 0.001). Gut microbiota: both ↓ Firmicutes, ↑ E. coli; sardine group specifically ↓ Firmicutes/Bacteroidetes ratio and ↑ Bacteroides-Prevotella. No significant differences in HbA1c between groups. Suggests cardiometabolic benefit more than glycemic benefit.
Eriksen et al., 2020 [21]	Randomized crossover trial (eight weeks)	40 men with a metabolic syndrome risk profile	Whole-grain rye diet ± lignan (secoisolariciresinol diglucoside, 280 mg at weeks 4–8)	Whole-grain wheat diet	Glucose tolerance (OGTT), cardiometabolic outcomes (lipids, BP), microbiota composition, enterolignans	WG rye (± lignan) did not improve glucose tolerance vs. wheat. After 4 weeks, rye ↓ total cholesterol (−0.06 mmol/L) and LDL-C (−0.09 mmol/L, P < 0.05), but effect not sustained at 8 weeks. WG rye ↑ Bifidobacterium (FC = 2.58, P < 0.001) and ↓ Clostridium genus (FC = 0.54, P = 0.02). Response to the WG diet differed by baseline microbiota enterotype.

Risk of Bias Assessment

The risk of bias assessment summarized in Table 2 indicates that most included studies demonstrated low risk across key domains such as randomization, outcome measurement, and completeness of follow-up, particularly in double-blind, placebo-controlled trials. However, open-label designs and short-duration crossover interventions introduced potential performance and carryover biases in some cases, while pilot or proof-of-concept studies raised concerns regarding selective reporting due to limited scope and complex outcome endpoints. Overall, although the evidence base is generally robust and methodologically sound, the identified concerns highlight the importance of cautious interpretation and emphasize the need for larger, well-blinded, multicenter studies to strengthen confidence in the observed associations.

**Table 2:** Summary of risk of bias assessment for randomized and dietary intervention trials This summary evaluates gut microbiota-targeted therapies in type 2 diabetes mellitus and related metabolic disorders. RCT: Randomized controlled trial, n: Number of participants, CGM: Continuous glucose monitoring, HbA1c: Glycated hemoglobin, BP: Blood pressure, CRP: C-reactive protein.

Authors (Year)	Randomization Process	Deviations from Intended Interventions	Missing Outcome Data	Measurement of the Outcome	Selection of the Reported Result	Overall Risk of Bias
Depommier et al., 2019 [12]	Low (randomization, blinding clear)	Low (double-blind, placebo)	Low (32/40 completed)	Low (objective metabolic outcomes)	Low	Low
Wang et al., 2022 [13]	Low (multicenter RCT, good concealment)	Low (double-blind, placebo-controlled)	Low (large n, minimal attrition reported)	Low (biochemical measures)	Some concerns (complex outcome reporting, many secondary endpoints)	Some concerns
Rein et al., 2022 [14]	Low (randomized crossover, well described)	Some concerns (short duration crossover may have carryover effect)	Low (high completion rate)	Low (CGM and HbA1c are reliable)	Low	Some concerns
Deng et al., 2022 [15]	Some concerns (open-label → performance bias risk)	High (not blinded, behavior may differ by treatment group)	Low (minimal attrition reported)	Low (objective lab outcomes)	Low	Some concerns
Companys et al., 2022 [16]	Low (double-blind RCT)	Low (good control/placebo)	Low (n=120, balanced arms)	Low (standard metabolic markers)	Low	Low
Kallapura et al., 2024 [17]	Some concerns (open-label design)	High (no blinding → intervention adherence bias)	Low (30/30 analyzed)	Low (HbA1c, BP, CRP are objective)	Some concerns (pilot “proof of concept” study → selective reporting possible)	Some concerns
Feng et al., 2022 [18]	Low (double-blind, placebo-controlled)	Low	Low (reasonable follow-up, maintenance phase)	Low (objective endpoints, neuropathy scoring validated)	Low	Low
Haro et al., 2016 [19]	Low (randomized, clear dietary allocation)	Some concerns (open-label diet trial, adherence self-reported)	Low	Low (microbiota + metabolome objectively measured)	Some concerns (dietary adherence reporting selective)	Some concerns
Balfegó et al., 2016 [20]	Low (randomized, dietary trial)	Some concerns (open-label nutrition trial, adherence issues)	Low (35/35 analyzed)	Low	Some concerns (pilot, small sample)	Some concerns
Eriksen et al., 2020 [21]	Low (crossover RCT, good design)	Some concerns (possible carryover effects)	Low	Low	Low	Some concerns

Discussion

Summary of Main Findings

This systematic review demonstrates that modulation of the gut microbiota through dietary, probiotic, pharmacological, and personalized nutrition strategies consistently yields favorable effects on glycemic control, insulin sensitivity, and cardiometabolic outcomes in patients with type 2 diabetes mellitus (T2DM) or at high metabolic risk. Pasteurized *Akkermansia muciniphila* improved insulin sensitivity by 28.6% and reduced insulinemia by 34% [12], while combined berberine and *Bifidobacterium breve* supplementation reduced postprandial LDL cholesterol more effectively than either agent alone [13]. Personalized machine learning-guided diets integrating microbiome profiles achieved diabetes remission in 61% of newly diagnosed patients and significantly reduced triglycerides by 49 mg/dL [14]. Pharmacological intervention with empagliflozin reshaped the microbiome-enriching butyrate-producing species such as Roseburia and Faecalibacterium, and uniquely improved cardiovascular markers beyond glycemic control [15]. Diet-based strategies, including seafood sticks enriched with heat-inactivated *Bifidobacterium animalis* and omega-3 [16], Mediterranean versus low-fat diets [19], and sardine-enriched diets [20], consistently linked microbial enrichment to improved insulin sensitivity and lipid metabolism. Collectively, these trials highlight the gut-metabolic axis as a therapeutic target, with interventions enhancing short-chain fatty acid producers such as Faecalibacterium prausnitzii and Bifidobacterium emerging as particularly beneficial.

Critical Comparison and Contrast

A critical evaluation of the included studies reveals heterogeneity in efficacy that appears to be driven by the nature of intervention, baseline disease stage, and host-microbiome interactions. For example, live versus pasteurized Akkermansia muciniphila produced divergent outcomes, with the pasteurized form demonstrating superior metabolic improvements, likely due to preserved membrane proteins stimulating host pathways [12]. In contrast, pharmacological modulation with empagliflozin induced rapid and broad microbial shifts toward butyrate-producing taxa, an effect not mirrored by metformin, underscoring drug-specific microbiome crosstalk [15]. Dietary interventions generally produced gradual but sustained microbial changes, such as the rise in Roseburia with the Mediterranean diet [19], while machine learning-based personalized diets achieved stronger glycemic responses than standardized Mediterranean diets, highlighting the role of individualized microbiome-host integration [14]. Notably, sex-specific effects emerged in [16], where women experienced a greater reduction in pulse pressure, suggesting hormonal or microbial compositional differences in responsiveness. Furthermore, heterogeneity in outcomes across whole-grain interventions indicated that baseline enterotype predicted cholesterol reduction [21], a finding that reinforces the emerging paradigm of precision nutrition. These contrasts emphasize that while microbiome modulation consistently benefits metabolic outcomes, the magnitude and nature of benefit depend on the form of intervention, host phenotype, and baseline microbial ecology.

Mechanistic Insights: Linking Microbiome Shifts to Clinical Benefit

The included RCTs collectively highlight how microbiome modulation mediates clinical improvements in T2DM through distinct biological pathways. Supplementation with pasteurized *Akkermansia muciniphila *[12] enhanced gut barrier integrity and reduced endotoxemia, directly improving insulin sensitivity by 28.6%. Pharmacological modulation with empagliflozin [15] enriched butyrate-producing taxa (Roseburia, Faecalibacterium, Eubacterium) while reducing harmful species such as Escherichia-Shigella, leading to improved cardiovascular markers beyond glycemic control. Personalized nutrition guided by machine learning algorithms [14,17] demonstrated that therapeutic responses are contingent on baseline microbial signatures, with precision diets tailored to microbiome composition driving sustained HbA1c reductions and diabetes remission in subsets of patients. Dietary strategies such as sardine-enriched or Mediterranean diets improved omega-3 indices, enriched anti-inflammatory taxa, and reduced pro-atherogenic microbial pathways, suggesting a dual role in both metabolic and cardiovascular protection [19,20]. These mechanistic links underscore that the gut-metabolic axis acts as a mediator rather than a passive bystander in T2DM, supporting the rationale for microbiome-targeted therapies.

Gaps and Limitations in the Literature

Despite promising findings, several limitations constrain translation into clinical practice. Many RCTs were short-term, typically lasting three to six months, leaving the sustainability of microbiome-induced benefits over years largely untested. Sample sizes were modest-often fewer than 120 participants-with some pilot trials including as few as 20 patients, limiting statistical power and external validity. Methodological inconsistencies, such as reliance on 16S rRNA sequencing in some studies versus whole-genome shotgun sequencing in others, hinder comparability across interventions. Outcomes were primarily surrogate endpoints, HbA1c, HOMA-IR, or lipid changes, whereas hard endpoints such as cardiovascular events, hospitalization, or diabetic complications were rarely examined, with the exception of neuropathy outcomes in the WCBE trial [18]. Moreover, most cohorts were ethnically homogeneous and geographically narrow, restricting insights into population-level variability in microbiome response. Finally, sex-specific differences were reported only incidentally, as in Companys et al., suggesting that stratified analyses by sex, age, and ethnicity remain underexplored [16]. Together, these gaps highlight the urgent need for larger, longer-duration, and methodologically harmonized trials that incorporate clinically meaningful endpoints and diverse populations.

Novel and Useful Insights

An important emerging insight from this synthesis is that microbiome profiling should be considered for integration into diabetes management algorithms, allowing clinicians to stratify patients into likely responders to diet-based versus pharmacological therapies. Evidence from Rein et al. [14] and Kallapura et al. [17] indicates that baseline microbial composition can predict therapeutic response, suggesting that a "precision nutrition" model could optimize outcomes and reduce trial-and-error in T2DM management. Furthermore, combination strategies appear more effective than monotherapies, as seen in Wang et al. [13], where berberine plus probiotics produced synergistic lipid-lowering and microbiome-mediated effects beyond either intervention alone. The role of microbiome-targeted therapies in mitigating complications is also noteworthy. Feng et al. [18] demonstrated improvement in diabetic neuropathy, while Deng et al. [15] linked microbial shifts to cardiovascular benefit, expanding the scope of microbiome modulation beyond glycemic control alone. Finally, artificial intelligence and machine learning models, exemplified by the personalized postprandial-targeting diet, show scalability for global application in diabetes care [14]. Yet, the stability of induced microbial shifts after stopping interventions-microbiota resilience-remains a largely unstudied dimension, representing a key knowledge gap with direct implications for long-term disease management.

Clinical Implications

The findings of this review suggest that microbiome-based interventions could represent a cost-effective and scalable adjunct to traditional T2DM therapies, particularly in resource-limited settings where access to novel agents such as SGLT2 inhibitors or GLP-1 receptor agonists is restricted [22]. Dietary strategies, such as whole grains, Mediterranean diets, or functional foods enriched with probiotics, are inexpensive, culturally adaptable, and could be incorporated into routine dietary counseling for diabetes patients. Moreover, gut microbial signatures-if validated-hold promise as predictive biomarkers for therapeutic success, guiding the choice of whether a patient is more likely to benefit from dietary modification, pharmacotherapy, or combined approaches [23]. By targeting the gut-metabolic axis, clinicians may not only improve glycemic control but also reduce the risk of microvascular and macrovascular complications, thereby reshaping the paradigm of holistic diabetes management.

Future Directions

To translate these promising results into practice, future research should prioritize large-scale, multicenter RCTs with long-term follow-up (>12 months) to assess the sustainability of microbiome-induced benefits and their impact on hard outcomes such as cardiovascular events, renal decline, or neuropathy progression. Integration of multi-omics platforms, including metabolomics, proteomics, and transcriptomics, will be essential to unravel causal pathways linking microbial changes with host metabolic physiology. Trials directly comparing personalized versus standardized microbiome-directed interventions are also warranted to determine the added value of precision strategies in real-world populations. Importantly, stratified analyses addressing sex-specific and age-specific responses to microbiome modulation must be incorporated, given signals of differential efficacy observed in some trials (e.g., blood pressure effects in women [16]). Finally, understanding the resilience of microbiota changes-whether interventions induce permanent or transient shifts-will be critical for designing long-term therapeutic protocols.

## Conclusions

This systematic review highlights the pivotal role of gut microbiota modulation in improving insulin resistance and mitigating complications in type 2 diabetes. The evidence from randomized controlled trials demonstrates that both nutritional and pharmacological strategies, ranging from supplementation with specific microbes and bioactive compounds to microbiome-informed personalized diets, can induce favorable shifts in microbial composition, increase short-chain fatty acid-producing taxa, and translate these changes into measurable metabolic benefits. While most studies were limited by short duration and modest sample sizes, the consistency of findings across diverse interventions underscores the potential of microbiome-targeted therapies as an adjunct to standard diabetes care. The significance of this review lies in its synthesis of clinical and mechanistic insights, providing a framework for integrating microbiome profiling into diabetes management and informing future large-scale, long-term trials. Ultimately, the gut-metabolic axis may represent a cornerstone in the move toward precision medicine in type 2 diabetes.
